# Prevalence and correlates of depression among older persons in rural eastern Uganda: a cross-sectional survey

**DOI:** 10.1080/16549716.2026.2682680

**Published:** 2026-06-05

**Authors:** Stephen Ojiambo Wandera, Peter Kisaakye, Shafiq Kawooya, Monica M. Diaz, Noeline Nakasujja

**Affiliations:** a Department of Population Studies, School of Statistics and Planning, College of Business and Management Sciences, Makerere University, Kampala, Uganda; b Department of Psychology & Neuroscience, University of North Carolina at Chapel Hill School of Medicine, Chapel Hill, NC, USA; c Department of Psychiatry, Makerere University, Kampala, Uganda; d Department of Psychiatry, School of Medicine, Makerere University College of Health Sciences, Kampala, Uganda

**Keywords:** Depressive symptoms, older adults, aged, Sub-Saharan Africa, Mental Health

## Abstract

**Background:**

Depression is one of the most common mental health disorders in developing countries, although it remains neglected. However, little is known about the prevalence and risk factors associated with depression among older persons living in rural communities in Uganda.

**Objective:**

To estimate the prevalence of depression and assess the factors associated with depression among older persons in rural communities in Uganda.

**Methods:**

We used cross-sectional data from two districts of Busia and Namayingo in rural eastern Uganda among 598 older persons aged 60 years and above. We generated descriptive statistics and chi-square tests to examine the differences between depression and the explanatory variables. Using a stepwise approach, we used multivariable logistic regression analyses to examine the association between depression and explanatory variables.

**Results:**

More than half of respondents (55%) reported moderate to severe depressive symptoms. Having moderate or severe depressive symptoms was associated with respondents who depend on their children for livelihood (OR = 1.84; CI = 1.02–3.32) or respondents who use firewood as a source of energy or lighting (OR = 2.72; CI = 1.43–5.16). Respondents who were living alone or socially isolated (OR = 2.05; CI = 1.35–3.12), fallen or at the risk of falling (OR = 2.05; CI = 1.35–3.12), frail (OR = 2.57; CI = 1.61–4.10) or being hypertensive (OR = 1.71; CI = 1.03–2.85) were more likely to report moderate or severe depressive symptoms than their counterparts.

**Conclusion:**

Depression remains more prevalent among older persons in low rural resource settings in Uganda. This underscores the need for targeted interventions that promote proper screening, treatment, service delivery, and better health-seeking behaviors among older persons in Uganda.

## Paper Context

- **Main findings:** More than half of older adults in rural eastern Uganda experienced moderate to severe depressive symptoms, with a higher risk among those who were frail, socially isolated and/or reliant on their children for support.
- **Added Knowledge:** This study provides new evidence from rural Uganda showing that late-life depression is closely linked to social-demographic, socio-economic and behavioral vulnerabilities underscoring the interplay between frailty, social isolation, and economic status.
- **Global health impact for policy and action:** Integrating depression screening and psychosocial support into targeted community-based geriatric care can strengthen healthy ageing policies and inform mental health strategies in low-resource settings.

## Background

Depression is one of the most common mental health disorders in developing countries, although it remains neglected [1,2]. Moreover, depression has been reported to be the leading cause of disability worldwide [3]. Depression among older persons remains a public health concern [4], with a significant proportion of older persons experiencing depressive symptoms [5]. In this paper, we use depression and depressive symptoms interchangeably. Following the Uganda National Council for Older Persons Act, we define an older person as anyone aged 60 years and above [6].

The World Health Organization (WHO) defines depression as a state of sadness, loss of interest, worthlessness, lack of sleep, lack of appetite, feeling tired, lack of concentration, or having a sense of guilt [7]. According to the WHO, approximately 280 million people worldwide suffer from depression, with 5% of adults suffering from depression [8]. In Uganda, it was estimated that 5.4% (representing 5269 per 100,000 people) in 2021 had a depressive disorder [9]. Depression remains one of the leading causes of death among older persons in sub-Saharan Africa (SSA) [10]. With an increase in the proportion of older persons in SSA, this is expected to almost double by 2050 [11,12]. Depression will be the leading contributor to the global burden of disease [13,14].

Although treatment regimens for depression are widely known, WHO observes that 75% of people with depressive disorders in low- and middle-income countries do not receive the necessary treatment, either due to poor diagnosis [10] or accessibility challenges [15] or limited specialists to diagnose [16] and treat depression [17], stigma [8]. Aging comes with health needs, although the management of such needs is often challenging [18].

Previous research has indicated that depression is associated with socio-demographic factors and health-related factors. A systematic review of the prevalence and determinants of depression documented these key risk factors [19]. The social-ecological systems theory guided this study. According to this theory, individual demographic and personal factors, and family-related factors, account for depression [20]. These include age, sex – being female [10], marital status, education level- low or no education [21], and religion. Health-related factors include morbidity, disability, and mortality [15]. Others include a poor quality of life [4,22], increased burden on public health utilization, poor socio-economic status [23,24], lack of social support [17], living alone or being isolated, suicidality [15,25,26], or people living with HIV [1].

Older persons with limited resources or no retirement savings often find themselves unable to get themselves food or basic needs, which increases stress and thereby leads to depressive symptoms [15,27]. Additionally, older persons are more likely to experience depressive disorders due to functional impairment [15,28–31] or withdraw from social engagements [32].

While previous research has indicated an increase in depressive disorders [13], depression among older persons in SSA in general and Uganda in particular remains under-researched in terms of the prevalence and risk factors [10,33,34]. Moreover, older persons in SSA often experience stress, worry or not being happy, even though they are likely to underreport depressive disorders [35]. Yet, these are common occurrences among older persons in Uganda, with 5% of the total population being 60 years and above [36].

While there is a vast body of knowledge among older persons in Uganda, most of these studies have focused on loneliness [37], health care access [18,37,38], quality of life [39–41], violence [42], HIV [43–46], or disability [47–49]. However, little is known about the prevalence and risk factors associated with depression among older persons living in rural communities in Eastern Uganda. Yet, not having up-to-date information about the prevalence and risk factors of depression may undermine coming up with strategies that may help with screening, treatment, and service delivery [50]. We bridge this gap by assessing the factors associated with depression among older persons in rural Eastern Uganda. Also, we integrate social and health correlates and provide implications for community-based screening of depression in rural Uganda.

## Methods

### Study design and setting

This was a cross-sectional survey covering two districts of Busia and Namayingo in rural eastern Uganda. The study sampled Busia and Namayingo because they have poor health and demographic indicators, including poverty, and limited access to healthcare for older persons [18]. They are also border districts near the border of Uganda and Kenya. They have mining activities that have escalated after 2019 [51,52]. These districts were selected out of 40 districts in the eastern Ugandan region [53,54].

### Sampling strategy and sample size calculation

A multi-stage stratified cluster sampling design was used to select households for the study. A sampling frame of households with older people was constructed from the 2014 Uganda Population and Housing Census sampling frame from the Uganda Bureau of Statistics [54,55]. In the first stage, two rural districts were randomly chosen from the administrative region of eastern Uganda. Specifically, the study sampled from the randomly chosen districts of Busia and Namayingo, which have poor health and demographic indicators, including poverty, and limited access to healthcare for older persons [18]. These districts were selected out of 40 districts in the eastern Ugandan region [53,54].

In the second stage, two out of ten sub-counties were chosen from each district using simple random sampling. In the third stage, computer-generated randomized numbers were used to select one parish from each sub-county. In the fourth stage, simple random sampling was used to select six enumeration areas. The sampling frame was then constructed for each village by consulting with local council leaders. Systematic sampling was used to select at least 30 households from each of the 24 villages that were included in the study. Household sampling continued until the enrollment goal was reached. This sampling frame was developed based on the 2014 Uganda Population and Housing Census sampling frame from the Uganda Bureau of Statistics [55]. A detailed sampling strategy is published elsewhere [56].

The prevalence of depression in Uganda is estimated at 22% from a systematic review and meta-analysis on the prevalence of mental disorders [57]. This, however, being part of a larger study on dementia, we used the dementia prevalence estimated at 20% from a cross-sectional study conducted in Uganda [58,59]. Using this prevalence of dementia (20%), the *p* = 0.20 and the q = 0.80. The level of confidence is 95% (z = 1.96), and the error was set at 5% (e = 0.05). The sample size calculated was 245. The sample size was multiplied by the design effect (D = 2). Thus, the sample size calculated was 492. The final sample size after adjusting for a response rate of 95% became 518. The overall sample size was 598 dyads of older adults and their caregivers. However, we surveyed 603 dyads of older adults and their caregivers. About five dyads could not be matched by RedCap and were therefore dropped from the analyses. Hence, we retained a sample of 598 dyads of older persons and their caregivers. For this paper, we analyzed data for older adults only [56].

### Data collection and recruitment of respondents

Data collection was conducted between December 2023 and September 2024. From each household that was selected, a dyad of an older adult and an in-home caregiver was interviewed. In households where older adults were living together as a couple, each older adult was interviewed separately. An older person was defined according to the United Nations’ criteria of age 60 years and older [60]. The prevalence of adults aged 60 years and older in Uganda in 2024 was 5% [53,54].

### Eligibility criteria

We included households in which at least one adult aged 60 years or older was available at the time of the home visit. Also, the older person must have lived in the selected household for at least 6 months, and could provide a written informed consent to participate in the study (if the person has the capacity to consent, or if not able to consent, then consent was obtained from a caregiver in the household). In addition, the caregiver needed to be present at the time of the interview with the older adult [56]. Both had to provide written consent to participate in the study. Older adults who could not write would make a thumbprint provided by the Research Assistants.

We excluded households in which no eligible household member could be interviewed due to a physical disability limiting ability to participate in cognitive testing (i.e. hearing or visual impairment) or other mental health condition, or those taking medications that could affect cognitive testing performance in the past 7 days (i.e. opiate or pain medication). Also, we excluded those whose caregivers were not present and could not be traced, even with phone calls for interview appointments [56]. About five older adults were not tested for height and weight because they could not stand up. For some who were frail, they were supported by the nurses and the caregivers to do height and weight assessments. These were not tested for the timed get-up-and-go test. This did not affect the survey that much.

### Ethical considerations

Ethical approval was obtained from the national Institutional Review Board – TASO Research Ethics Committee (Approval Number: TASO-2022–179) and the Uganda National Council of Science and Technology (UNCST). The approval number is HS2693ES. Voluntary informed consent was obtained from all study participants (both the older adult and the caregiver in each household). The consent to participate was written – older adults who would not write would use a thumbprint to endorse consent forms after it was read to them in the local languages (Lusamia or Lusoga). Details are published elsewhere [56]. Our study was carried out in accordance with the principles of the Declaration of Helsinki.

### Variables and measures

#### Outcome variables: depression

Depression screening was completed using the 9-item Patient Health Questionnaire (PHQ-9) [61–64], which has been previously applied in Uganda [64]. A score of 10 or greater was used to define moderate to severe depression on the PHQ-9 [64]. The PHQ-9 indicators had a Cronbach’s alpha of 0.85.

#### Explanatory variables

The choice of explanatory variables was informed by the systematic review and meta-analysis about the prevalence and determinants of depression in old age [19] and the social-ecological systems theory [20]. The explanatory variables included biosocial (demographic and socio-economic factors), behavioral, and health-related factors. Demographic variables included sex (male and female), age group, and number of children surviving. Socio-economic variables included: educational level, working status, source of livelihood, source of energy for household lighting, marital status, and religion.

Behavioral variables include smoking and alcohol consumption. We asked participants if they currently or formerly smoke(d) cigarettes (yes or no). Possible alcohol use disorder was measured using the CAGE assessment for alcohol use disorder risk [65–67], previously applied in Uganda [66,67]. The CAGE has four questions that include:

1. Do you drink alcohol?
2. Have you ever felt you should cut down on your drinking?
3. Have people annoyed you by criticizing your drinking of alcohol?
4. Have you ever felt bad or guilty about your drinking?

Their responses are yes and no. The Cronbach’s alpha for the CAGE scale was 0.60.

Falls were measured by combining two questions (with yes and no responses) on the experience of falls and the fear of the risk of falling in the future. These questions were:

1. Have you ever fallen in the last 6 months?
2. Do you think you may fall in the next few months?

We combined any response to either of the two questions as an indicator of falls.

We also measured frailty using the FRAIL scale [68–72]. Frailty has five questions about fatigue, resistance, aerobics, illness, and loss of weight [72]. A score of 3 to 5 indicates frailty, and 0 to 2 indicates no frailty [72].

We measured social isolation and living alone using three questions (yes and no):

1. Do you live alone?
2. Are you unable to go outside the home?
3. Are you able to participate in group activities?

Social isolation and living alone were created by recoding a yes to any of the three questions.

Loneliness was assessed using the UCLA Loneliness Scale – Short Form [73–75]. The six questions are:

1. I lack companionship.
2. I feel part of a group of friends.
3. I feel left out.
4. I feel isolated from others.
5. I am unhappy being so withdrawn.
6. People are around me but not with me.

Their responses range from 1 to 4. Item 2 was reverse coded so that it is negative. The loneliness scores range from 6 to 24, with higher scores indicating greater loneliness. The Cronbach’s alpha for the CAGE scale was 0.76. The mean loneliness score was 12.5 (standard deviation (SD) 4.3, minimum 6, maximum 24).

To screen for dementia, we used the Identification and Intervention for Dementia in Elderly Africans (IDEA) cognitive screening tool [76,77]. The IDEA tool is a brief cognitive screening tool validated in rural Tanzania for older adults with low literacy and has demonstrated high diagnostic accuracy for dementia. The tool has been validated in other sub-Saharan African populations, including rural community samples of older adults in Tanzania and Nigeria, with normative values established among these populations [76,77]. The IDEA is scored from 0 to 15, including assessments of abstraction, orientation, long-term memory, categorical verbal fluency, visuospatial construction, and verbal delayed recall. The tool does not require the ability to read, write, draw, or calculate. An external validation study in Tanzania established the following cut-off scores, which we applied for our study: 0–7 ‘probable dementia’; 8–9 ‘possible dementia’; and 10–15 ‘no dementia’ [77].

We also measured the following during each assessment of the older adult: blood pressure, weight, and height for body mass index. Hypertension was defined as SBP 140 mm Hg or higher and DBP 90 mm Hg or higher [78,79]. Hyperglycemia was assessed using a random blood glucose test performed during the assessment and defined as random blood glucose ≥ 200 mg/dL.

### Statistical analysis

All survey data was entered into RedCap hosted at Makerere University, College of Health Sciences. Redcap is a secure data collection platform which does not collect personal information.

For all descriptive statistics, we used frequency distributions for categorical variables and mean and standard deviations (if normally distributed) or median and interquartile range (if not normally distributed) for continuous variables (e.g. loneliness and PHQ-9). We used cross-tabulations to investigate associations between depression (binary outcome: none or mild depression vs moderate to severe depression) and selected explanatory variables. Pearson’s chi-squared (χ2) tests were used to examine the differences between depression and the explanatory variables. The level of statistical significance using *p*-values was set at *p* < 0.05.

We used multivariable logistic regression analyses to examine the association between depression and explanatory variables whose *p*-values were less than 0.05 during the chi-square tests. For those whose *p*-values were higher, we determined to add them a priori based on the literature [80]. We used stepwise regressions for multivariable analysis, including all relevant covariates following the Biopsychosocial model for depression [80]. However, our data had biological factors such as age, gender, health conditions and chronic illness, and physical function and social factors (e.g. age, sex) but did not include psychological factors [80,81]. First, in Model 1, we adjusted for biosocial factors first (demographic and socio-economic variables). Finally, in Model 2, we adjusted behavioral and health factors, including hypertension status, suspected alcohol use disorder, smoking, and loneliness. We present results in the form of Odds Ratios (OR) reporting 95% confidence intervals. The level of statistical significance using *p*-values was set at *p* < 0.05. All analyses were performed in STATA version 19.

## Results

### Severity of depression

**Figure 1.**
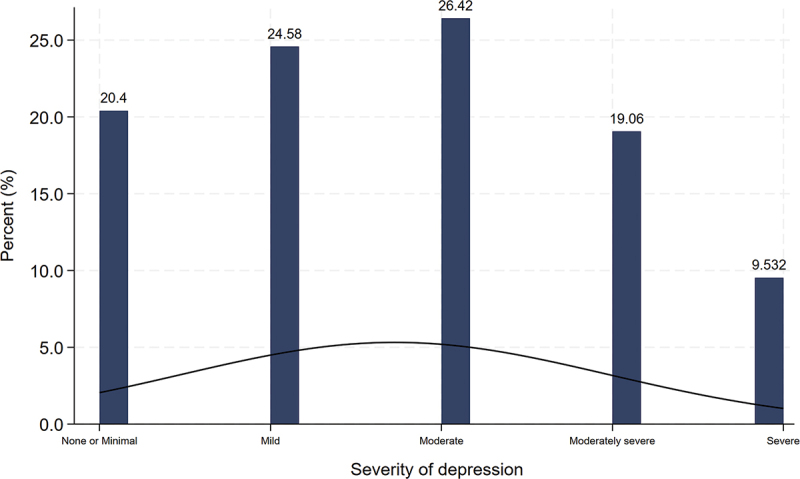
Severity of depression.

Figure 1 shows the severity of depression in the study population. About two out of every ten respondents (20.4%) had none or minimal depression. Most respondents had moderate depression (26.4%), and the least proportion of respondents had severe depression (9.5%).

### Relationship between depression and the age of respondents

**Figure 2.**
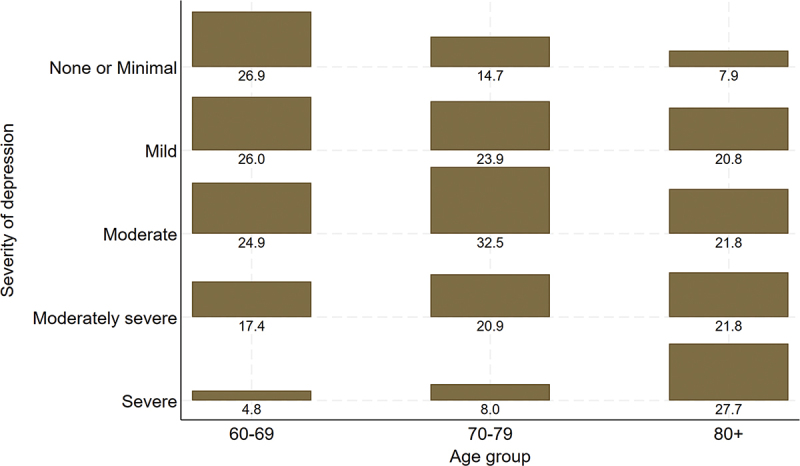
Depression severity by age group.

Figure 2 shows the relationship between depression and the age groups of respondents. Results indicate that age is directly related to the experience of severe depression. That is, as age increases, the proportion of people who experience severe depression also increases. On the other hand, age was inversely related to none or minimal depression. That is, as age increases, the proportion of people with no or minimal depression decreases. More younger people (60–69 years) had none or minimal depression (26.9%) compared to older people aged 80 years or older (7.9%).

### Descriptive characteristics of respondents by depression status

Table 1 shows the distribution of respondents in the study by depression status. Overall, older persons had an average of six living children (standard deviation (SD = 4 children). The average loneliness score was 12.507 (SD = 4.343). The average timed up and go test was 15.466 (SD = 10.162). The average hand grip strength was 18.856 (SD = 7.202). The average Simple Nutritional Appetite Questionnaire (SNAQ) score was 13.22 (SD = 2.70).

**Table 1.** Descriptive characteristics of respondents by depression status.

	Experienced depression			
Variables	None or mild depression, n(%)	Moderate or severe Depression, n(%)	Total, n(%)	*p value*
Total (N)	269 (45.0)	329 (55.0)	598 (100.0)	
How many living children do you have?	6.996 (5.460)	5.883 (3.588)	6.384 (4.557)	0.003
UCLA Loneliness Scale – Short Form (ULS-6)	10.543 (3.854)	14.112 (4.055)	12.507 (4.343)	<0.001
Timed Up and Go (TUG) Test	13.839 (7.766)	16.796 (11.607)	15.466 (10.162)	<0.001
Average handgrip strength	20.494 (7.123)	17.517 (6.996)	18.856 (7.202)	<0.001
Simple Nutritional Appetite Questionnaire (SNAQ) score	13.792 (2.274)	12.757 (2.925)	13.222 (2.700)	<0.001
**Sex**				
Female	144 (53.5)	219 (66.6)	363 (60.7)	0.001
Male	125 (46.5)	110 (33.4)	235 (39.3)	
**Age group**				
60–69	177 (65.8)	157 (47.7)	334 (55.9)	<0.001
70–79	63 (23.4)	100 (30.4)	163 (27.3)	
80+	29 (10.8)	72 (21.9)	101 (16.9)	
**Marital status**				
Formerly married	137 (50.9)	217 (66.0)	354 (59.2)	<0.001
Currently married	132 (49.1)	112 (34.0)	244 (40.8)	
**Number of children alive**				
0–6 children	153 (56.9)	206 (62.6)	359 (60.0)	0.154
7–50 children	116 (43.1)	123 (37.4)	239 (40.0)	
**Education level**				
No education	82 (30.5)	150 (45.6)	232 (38.8)	<0.001
Primary or higher	187 (69.5)	179 (54.4)	366 (61.2)	
**Religion**				
Catholic	101 (37.5)	142 (43.2)	243 (40.6)	0.190
Anglican	74 (27.5)	94 (28.6)	168 (28.1)	
Pentecostal or others	94 (34.9)	93 (28.3)	187 (31.3)	
**In the last 12 months, have you done any work for which you received a payment**				
No	205 (76.2)	227 (69.0)	432 (72.2)	0.050
Yes	64 (23.8)	102 (31.0)	166 (27.8)	
**Source of livelihood**				
Farming	195 (72.5)	201 (61.1)	396 (66.2)	<0.001
Depend on children	36 (13.4)	85 (25.8)	121 (20.2)	
Trading or others	38 (14.1)	43 (13.1)	81 (13.5)	
**Source of energy for lighting**				
Electricity or solar	165 (61.3)	141 (42.9)	306 (51.2)	<0.001
Paraffin lamp	74 (27.5)	92 (28.0)	166 (27.8)	
Firewood or others	30 (11.2)	96 (29.2)	126 (21.1)	
	**Experienced depression**			
Variables	None or mild depression, n(%)	Moderate or severe Depression, n(%)	Total, n(%)	*p value*
Total (N)	269 (45.0)	329 (55.0)	598 (100.0)	
**Alcohol problems using the CAGE scale**				
No	183 (68.0)	244 (74.2)	427 (71.4)	0.099
Yes	86 (32.0)	85 (25.8)	171 (28.6)	
**Ever or currently smoking**				
No	245 (91.1)	282 (85.7)	527 (88.1)	0.044
Yes	24 (8.9)	47 (14.3)	71 (11.9)	
**Living alone and socially isolated**				
No	171 (63.6)	121 (36.8)	292 (48.8)	<0.001
Yes	98 (36.4)	208 (63.2)	306 (51.2)	
**Ever fallen or at risk of falling**				
No	124 (46.1)	97 (29.5)	221 (37.0)	<0.001
Yes	145 (53.9)	232 (70.5)	377 (63.0)	
**Frailty status**				
Not frail	223 (82.9)	170 (51.7)	393 (65.7)	<0.001
Frail	46 (17.1)	159 (48.3)	205 (34.3)	
**Dementia by IDEA Tool**				
No dementia	234 (87.0)	241 (73.3)	475 (79.4)	<0.001
Probable dementia	35 (13.0)	88 (26.7)	123 (20.6)	
**Systolic BP > 140 mmHg and diastolic BP > 90 mmHg**				
Normal BP	225 (83.6)	246 (74.8)	471 (78.8)	0.008
Hypertensive	44 (16.4)	83 (25.2)	127 (21.2)	
**Possible diabetes based on random blood glucose level**				
No diabetes	251 (93.3)	300 (91.2)	551 (92.1)	0.337
Possible diabetes	18 (6.7)	29 (8.8)	47 (7.9)	

Six in ten (61%) older persons were older women, and 56% were aged 60–69 years. About 41% were currently married, 60% had 0–6 children, and 39% had no education. Four in ten (41%) were Catholics, and only 28% had received payment from work done in the last 12 months. More than half (66%) were farmers, and slightly more than half (51%) used electricity or solar for lighting. Over a quarter (29%) had alcohol problems and one in ten (12%) were smokers. Living alone and social isolation was 51% and falls was 63%. A third (34%) were frail and 21% had probable dementia. About 21% were hypertensive and 8% had probable diabetes.

### Cross-tabulations of depression and covariates

Table 1 shows the cross-tabulations of depression and selected covariates. Most (55%) respondents had moderate to severe depression. There were significant differences observed between respondents with none or mild depression and those with moderate to severe depression in terms of sex, age, education, source of livelihood, source of energy for lighting, marital status, smoking status, risk of falling, frail status, loneliness status, dementia status, and blood pressure status.

Experience of moderate or severe depression was associated with having fewer living children (5.9% vs 6.9%) and loneliness (14.1% vs 10.5%) compared to their counterparts. Respondents with a longer time to get up and go test (in seconds) had moderate or severe depression compared to their counterparts with less time (16.8% vs 13.8%). Experience of moderate or severe depression was associated with less handgrip strength (17.5% vs 20.5%) and poor nutrition (12.7% vs 13.7%) compared to their counterparts with none or minimal depression.

Six out of ten older women (66.6%) had moderate to severe depression compared to a third of older men (33.4%). More respondents who were younger, aged 60–69 (47.7%), with primary or higher education (54.4%), or engaged in farming (61.1%) had moderate or severe depression compared to their counterparts. Experience of moderate or severe depression was observed more among respondents whose source of energy for lighting is electricity or solar (42.9%), formerly married (66.0%), never smoked (85.7%), had a risk of falling (70.5%), or lived alone (63.2%) compared to their counterparts.

### Multivariate results

Table 2 shows results from logistic regression models assessing the factors associated with depression. Model 1, controlled for biosocial factors only. It shows that older persons aged 70–79 years (OR = 1.65; CI = 1.09–2.48), those who depend on their children for a source of livelihood (OR = 2.34; CI = 1.43–3.82), and those who used firewood as a source of energy for lighting (OR = 3.83; CI = 2.21–6.63), had increased odds of reporting moderate or severe depressive symptoms.

**Table 2.** Regression of depression against selected socio-demographic and health measures.

	Model (1)		Model (2)	
Variables	Adjust for Biosocial variables	95% Confidence Intervals (95% CI)	Adjust for behavioral and health factors	95% CI
**Sex**				
Female	1.00		1.00	
Male	0.78	[0.48–1.28]	0.79	[0.41–1.53]
**Age group**				
60–69	1		1	
70–79	1.65^*^	[1.09–2.48]	1.49	[0.91–2.42]
80+	1.62	[0.94–2.80]	0.96	[0.48–1.92]
**Marital status**				
Formerly married	1.00		1.00	
Currently married	0.83	[0.51–1.36]	1.32	[0.73–2.37]
**Number of children alive**	1.26	[0.85–1.85]	1.48	[0.95–2.32]
**Education level**				
No education	1.00		1.00	
Primary or higher	0.66	[0.43–1.01]	0.83	[0.49–1.40]
**Religion**				
Catholic	1.00		1.00	
Anglican	1.09	[0.71–1.68]	1.00	[0.61–1.64]
Pentecostal or others	0.76	[0.50–1.16]	0.69	[0.41–1.15]
**In the last 12 months, have you done any work for which you received a payment**	1.29	[0.81–2.06]	1.60	[0.94–2.73]
**Source of livelihood**				
Farming	1.0		1.00	
Depend on children	2.34^***^	[1.43–3.82]	1.84^*^	[1.02–3.32]
Trading or others	1.19	[0.71–2.01]	0.98	[0.53–1.80]
**Source of energy for lighting**				
Electricity or solar	1.00		1.00	
Paraffin lamp	1.31	[0.87–1.97]	1.00	[0.62–1.63]
Firewood or others	3.83^***^	[2.21–6.63]	2.72^**^	[1.43–5.16]
**Alcohol problems using the CAGE scale**			0.82	[0.50–1.35]
**Ever or currently smoking** No Yes			1.00 1.81	[0.93–3.50]
**Living alone and socially isolated** No Yes			1.00 2.05***	[1.35–3.12]
**UCLA Loneliness Scale – Short Form (ULS-6)**			1.20^***^	[1.14–1.27]
Average handgrip strength			1.00	[0.97–1.04]
**Ever fallen and at risk of falling** No Yes			1.00 2.05***	[1.35–3.12]
**Frailty status**				
No			1.00	
Yes			2.57^***^	[1.61–4.10]
**Dementia by IDEA Tool**				
No dementia			1.00	
Probable dementia			1.24	[0.70–2.19]
**Systolic BP > 140 mmHg and diastolic BP > 90 mmHg**				
Normal BP			1.00	
Hypertensive			1.71^*^	[1.03–2.85]
**Possible diabetes based on random blood glucose level**				
No diabetes			1.00	
Possible diabetes			1.17	[0.55–2.50]
**Observations**	**598**		**598**	

Exponentiated coefficients; 95% confidence intervals in brackets; *p < 0.05, **p < 0.01, ***p < 0.001.

Model 2 adjusted for all socio-demographics and health measures. When we added behavioral and health factors to the model, the effect size reduced for respondents who depended on their children as a source of livelihood (OR = 1.84; CI = 1.02–3.32). The age group lost its significant association. Older persons who used firewood as a source of energy for lighting (OR = 2.72; CI = 1.43–5.16) had increased odds for depressive symptoms, even though the effect sizes were decreased. Respondents who were living alone or socially isolated (OR = 2.05; CI = 1.35–3.12), had fallen or were at the risk of falling (OR = 2.05; CI = 1.35–3.12), were frail (OR = 2.57; CI = 1.61–4.10), or were hypertensive (OR = 1.71; CI = 1.03–2.85), were more likely to report moderate or severe depressive symptoms.

This paper broadens the current body of knowledge regarding depression or depressive symptoms among older persons in rural eastern Uganda. Our findings show that slightly more than half (55%) of older persons had moderate or severe depressive symptoms, slightly lower than what was reported in a systematic review of depression prevalence in Uganda at 68% among refugees [57]. This prevalence was higher than what has been reported in other studies. A study in urban Uganda – Wakiso district reported about 19% of depression in a sample of 114 older persons [82]. However, this sample covered younger adults aged 35 years and older. The possible reason for the high prevalence of depressive symptoms is the presence of multiple health challenges (social isolation was 51%, frailty was 34% and experiencing falls was 63%) and non-communicable diseases (NCDs), yet with limited access to healthcare in the public sector [18]. More than half of the older adults were socially isolated. Older persons get stressed and worried about health problems, including NCDs, yet with no or limited access to medication and treatment [82]. Depression among older persons in rural eastern Uganda was associated with depending on children as a source of livelihood, using firewood as a source of energy, social isolation, loneliness, falls, frailty, and hypertension.

The likelihood of experiencing moderate or severe depressive symptoms was observed to be higher among respondents whose source of livelihood was their children. Previous research has reported that depression is likely to be higher among respondents who rely on their children. There is psychological and economic stress that comes with over-reliance on children for survival. Older persons who rely on their children, who may be economically stressed, may miss the emotional support that could come from parent-child interactions [23]. Alternatively, they experience increased insecurity or financial strain, which may further worsen the risk of depression [24]. More than half of the adults in rural eastern Uganda report higher poverty levels [82–84]. Therefore, depending on children exposes older persons to the risk of stress and emotional challenges.

The odds of depression were increased among older adults whose source of energy for lighting was firewood. As already stated, using firewood is an indicator of poverty. In our study, most older adults depend on their adult children. This is because more than half of the older persons either had no education or at most had a primary education. Therefore, most older persons have not worked in the public sector and therefore lack social protection or income security in old age. Most of them do not receive pensions for that reason. Also, older adults who are already frail find it difficult to locate firewood [84]. Finally, the use of firewood increases the risk of pollution, which is a risk factor for poor mental health outcomes [85,86].

Our analysis found that the experience of moderate or severe depressive symptoms was likely to occur among respondents who were living alone or socially isolated or experienced loneliness. This is likely due to the limited social interactions that may exacerbate loneliness, poor social support, or emotional support [25,26]. Living alone for older persons in a context of an extended kinship network is stressful [87]. It could be an indicator of ageism or loss of loved ones or children due to the HIV pandemic. Worrying and deep thoughts can kill! [88]. Even though we adjusted for both social isolation and loneliness, we used different scales to measure them. Social isolation is physical and relational. Loneliness includes overlapping measures of social isolation. After creating the indices for each of them, we tested for collinearity and pairwise correlations, and they had no collinearity. The revised UCLA loneliness scale is a validated measure [73,74,89,90].

Health variables exacerbate the risk of depression. These included physical falls, frailty, and hypertension. Falls or the risk of falls increased the odds of moderate or severe depressive symptoms. Previous studies have observed that depression tends to be related to mobility challenges, which undermine the balance necessary for preventing falls [29–31]. Also, our results show that frailty was strongly associated with depressive symptoms. Frailty involves physical vulnerabilities, including weight loss, being slow, reduced physical activity, or reduced muscle strength [28]. Frailty limits mobility and leads to heightened social isolation and loneliness. Finally, hypertension was associated with depressive symptoms. The challenge with hypertension is the absence of medications in the public health sector, especially at health center IIIs. Most older persons cannot afford to purchase them from private facilities. This makes them worry about their healthcare [18,91,92].

### Strengths and limitations

The strengths of the study include the following: First, it adds to the available literature that examines the socio-demographic and health-related factors for depression among older persons in a rural setting of eastern Uganda. Second, the use of a standard PHQ-9 validated tool to measure depression allows for the generation of robust estimates for comparability.

However, some limitations are worth highlighting. First, the cross-sectional data does not allow for causality assessment. The analysis uses cross-sectional data that may limit cause-and-effect relationships. Second, the cultural sensitivities, including stigma surrounding poor mental health outcomes in low-resource settings, can lead to social desirability bias, which leads to under-reporting. Third, whereas our focus is limited to a rural community in eastern Uganda, we are unable to generalize our findings to other settings. Fourth, we were unable to conduct additional analysis that incorporates the coping mechanisms for people experiencing depressive symptoms, which are important for guiding diagnosis, treatment, and service delivery. Fifth, although we used a multi-stage cluster sampling design, the analysis did not account for clustering or survey design effects, which may have implications for variance estimation and statistical inference. We did not generate and use survey weights to account for the complex survey design.

Finally, even though we used a multi-stage cluster sampling design, our analysis did not account for clustering or survey design effects (e.g. through cluster-robust or survey-adjusted methods.) We did not have survey weights to use in the data. Therefore, the interpretation of our estimations, findings, and conclusions needs to consider this limitation.

## Conclusion

Depression was more prevalent among older persons in low rural resource settings in eastern Uganda. Depression among older persons in rural eastern Uganda was associated with depending on children as a source of livelihood, using firewood as a source of energy, social isolation, loneliness, falls, frailty, and hypertension. The findings underscore the need for targeted interventions that promote proper screening, treatment, service delivery, and better health-seeking behaviors among older persons in Uganda.

Understanding the prevalence and correlates of depression among older people offers insights that are important for guiding strategies designed to promote depression screening, treatment, and service delivery among older adults in rural settings in Uganda. There is a need for the government of Uganda, through the Ministry of Health, to target screening and treatment of depression among older persons in rural Uganda.

## Supplementary Material

Reporting Guidelines_Checklist_STROBE_checklist_26122025.docx

## Data Availability

Data are available upon reasonable request from the authors.

## Strobe Checklist

Zenodo: https://zenodo.org/records/17326852
